# Evaluating Tranexamic Acid's Role in Upper Eyelid Blepharoplasty: A Systematic Review and Meta-Analysis

**DOI:** 10.1007/s00266-025-05294-w

**Published:** 2025-10-28

**Authors:** Mohamed Abo Zeid, Kareem Khalefa, Mohammad Al Diab Al Azzawi, Amr Elrosasy, Amr M. Abou Elezz, Mohamed S. I. Mohamed, Richard C. Allen, Hashem Abu Serhan

**Affiliations:** 1 Faculty of Medicine, Tanta University, Tanta, Egypt; 2 Faculty of Medicine, National Ribat University, Khartoum, Sudan; 3 Faculty of Medicine, Cairo University, Cairo, Egypt; 4 College of Medicine, QU Health, Qatar University, Doha, Qatar; 5 Department of Ophthalmology, Dell Medical School, University of Texas, Austin, TX US; 6 Department of Ophthalmology, Hamad Medical Corporation, Doha, Qatar

**Keywords:** Blepharoplasty, Tranexamic acid, Plasminogen, Bleeding, Oculoplastic

## Abstract

**Introduction:**

To evaluate the efficacy of tranexamic acid (TXA) in blepharoplasty surgery.

**Methods:**

A meta-analysis was conducted following PRISMA guidelines. PubMed, Web of Science, Cochrane Library, and Scopus databases were searched for randomized controlled trials (RCTs). Data extraction focused on outcomes, such as ecchymosis scores, time to recovery, intraoperative pain, operative time, and hemostasis quality. The risk of bias was assessed using the ROB 2 tool. RevMan 5.4 software was used for meta-analysis with mean differences (MD) and standardized MD (SMD) calculated for various outcomes.

**Results:**

Five RCTs comprising 594 participants (1154 eyes) met the inclusion criteria. Overall, TXA significantly reduced postoperative ecchymosis on postoperative days (POD) 1 and 7 (SMD = – 0.54, 95% CI: [– 0.71 to – 0.38], *p* < 0.00001) with moderate heterogeneity (*I*^2^ = 50%). TXA significantly shortened patient-reported recovery time (MD = – 0.72 days, 95% CI: [– 1.02 to – 0.42], *p* < 0.00001) with homogeneity (*I*^2^ = 0%). Also, TXA significantly reduced operative time (MD = – 1.20 minutes, 95% CI: [– 2.39 to – 0.01], *p* < 0.05). On the other hand there was no significant difference in intraoperative pain scores or surgeon-assessed hemostasis quality for SC TXA; however, IV TXA improved hemostasis assessment (MD = – 0.70, 95% CI: [– 1.02 to – 0.38], *p* < 0.0001).

**Conclusion:**

TXA significantly reduces ecchymosis, expedites recovery, and enhances operative time and hemostasis in blepharoplasty. These findings support TXA’s use as an effective adjunct to improve blepharoplasty recovery, although further studies are warranted to optimize dosing and minimize thromboembolic risks.

**Level of Evidence I:**

This journal requires that authors assign a level of evidence to each article. For a full description of these Evidence-Based Medicine ratings, please refer to the Table of Contents or the online Instructions to Authors www.springer.com/00266.

**Supplementary Information:**

The online version contains supplementary material available at 10.1007/s00266-025-05294-w.

## Summary Points

- TXA significantly reduced postoperative ecchymosis on both postoperative day 1 and 7, demonstrating consistent benefit across time points (SMD = – 0.54; *p* < 0.00001), with benefit remaining robust across sensitivity analyses and regardless of eyelid surgery type.
- TXA shortened patient-reported recovery time by nearly 0.72 days, indicating faster return to daily activities with no observed heterogeneity.
- IV TXA was superior to SC TXA in reducing operative time and improving surgeon-assessed hemostasis, though SC TXA still conferred some benefits.
- No significant effect was found on intraoperative pain scores across all included studies and routes of TXA administration.
- High-quality evidence supports TXA use in blepharoplasty based on GRADE assessment, though further studies are needed to optimize dosing and assess long-term safety.

## Introduction

Blepharoplasty is one of the most common oculoplastic surgeries carried out globally for both functional and cosmetic indications [1]. It is used to treat periorbital aging and dermatochalasis [2, 3]. Although postoperative satisfaction rates are generally high, the highly vascular periorbital region may result in challenging postoperative recovery. Ecchymosis, hematomas, and displeasing swelling can occur, leading to alarming cosmetic concerns and prolonged recovery. Along with discontinuing anticoagulants before surgery, which may increase thromboembolic complications in some patients, multiple studies have been carried out to optimize postoperative hemostasis [4, 5]. Cryotherapy (ice compress) is the most used method to control postoperative hemostasis; however, it has been shown to reduce pain rather than ecchymosis, hematoma, or swelling [6]. Preoperative control of hypertension is also essential to minimize postoperative ecchymosis along with the cessation of aspirin and anticoagulants [7]. Furthermore, the combination of Arnica and bromelain [8], magnesium sulfate wet dressings [9], and intense pulsed light (IPL) [10] have all been explored, but results remain inconsistent. In patients on antithrombotic medications, intraoperative fibrin glue has been shown to reduce postoperative ecchymosis [11].

Tranexamic acid (TXA) is a synthetic lysine analog that inhibits plasminogen activation to plasmin, by competitively blocking the binding sites on plasminogen molecules. This results in inhibition of plasmin degradation of fibrin clots [12, 13], which reduces bleeding and promotes stable clot formation [14]. Many studies have shown that TXA inhibits plasmin-induced platelet activation, promoting coagulation and its anti-inflammatory effects due to plasmin blockage [12, 15] Plasmin is also known to activate interleukin-6, which plays a major role in inflammatory reactions. Interleukin-6 levels increase 30–60 minutes after surgery, especially in vascular operations, reach their peak around 24 hours, and remain elevated for 48–72 hours postoperatively [16]. By inhibiting plasmin production, TXA contributes to the reduction of postoperative swelling.

TXA is used for women with excessive menorrhagia and significantly reduces transfusion requirements in patients undergoing total knee arthroplasty [13] and cardiac surgery [13, 15]. It also enhances intraoperative visualization of the surgical field by reducing blood loss, especially when used alongside epinephrine [17].

Although the recommended dosage and mode of administration of perioperative TXA are still open to debate, a large randomized controlled study indicated that all routes of administration within the given doses are safe and significantly reduce mortality [18]. TXA has shown significant potential for minimizing blood loss and associated postoperative hematomas and ecchymosis in plastic surgery and dermatological surgery [19, 20]. Nevertheless, the clinical benefits of TXA need to be weighed against the potential risk of thromboembolic events [21]. A study conducted by Knowlton et al [22] reported a higher incidence of thromboembolic events in trauma patients receiving intravenous (IV) TXA; however, another study conducted by Scarafoni et al [23] showed TXA to be effective in reducing blood loss with no increased risks of thrombotic events regardless of the route of administration. In this meta-analysis, we aimed to investigate the efficacy outcomes of administering TXA before blepharoplasty surgeries to evaluate its potential as a standard treatment in blepharoplasty practice.

## Materials and Methods

### Protocol Registration

This study was carried out and reported according to the Preferred Reporting Items for Systematic Reviews and Meta-Analysis (PRISMA) and the Cochrane Handbook for Systematic Reviews [24, 25]. This study was prospectively registered on Open Science Framework (OSF) with a registration number: 10.17605/OSF.IO/4QEBW.

### Search Strategy and Data Sources

Several key databases were searched from inception till October 2024 including PubMed, Web of Science (WOS), Scopus, and the Cochrane Library. Our search strategy was (“Tranexamic Acid” OR Cyklokapron OR Ugurol OR Transamin OR AMCA OR Anvitoff OR "Tranexamic Acid"[Mesh]) AND (Blepharoplasty OR Blepharoplasties OR Eyelid OR “eyelid surgery”). Rayyan software [26] was used to remove duplicates, and all retrieved publications were assessed for eligibility. Two reviewers [M.D.A. and K.K.] independently included articles if they fulfilled the criteria, and any disagreements in judgments were addressed by discussion.

### Eligibility Criteria

The inclusion criteria were established according to the PICOS framework: Population: patients undergoing blepharoplasty; Intervention: SC or IV TXA; Comparison: placebo or no TXA; Outcomes: ecchymosis score, patient-reported recovery time (days required to resume daily activities), intraoperative pain score, operative time, and surgeon's assessment of hemostasis; Study design: limited to randomized controlled trials (RCTs) only.

The exclusion criteria included animal studies, case reports, case series, commentaries, reviews, editorials, non-English studies and studies with only an abstract or unavailable full text. Studies with overlapped data or without a comparison group also were excluded.

### Study Selection and Data Extraction

Two independent reviewers [M.A. and A.A.] conducted data extraction using a standardized excel form taking baseline information such as country of publication, research design, demographic characteristics, follow-up periods, interventions (route of TXA administration, dose, and frequency), and efficacy and safety outcomes related to the use of TXA in blepharoplasty. Any differences throughout the selection or extraction process were handled by discussion.

### Quality Assessment

We used the Risk of Bias 2 (ROB 2) tool [27] to assess the quality of included articles in this review. The tool examines possible bias across five critical areas: the randomization process, deviations from the intended interventions, missing data, outcome measurement, and the selection of reported results. Each study was graded as having either low risk, some concerns, or a high risk of bias in each category. Two reviewers [M.D.A. and K.K.] independently completed these assessments, and any disagreements in judgments were addressed by discussion.

### Data Synthesis and Statistical Analysis

Review Manager (RevMan) version 5.4.1 was used to conduct the meta-analysis. Since all the outcomes were continuous, they were grouped into intraoperative and postoperative measures. Preoperatively, the intraoperative pain score was analyzed using the visual analogue scale (VAS), the duration of surgery in minutes, and the surgeon’s judgment of hemostasis on a 1–4 scale. Postoperatively, ecchymosis scores were analyzed at several time points immediately after surgery, on postoperative day 1 (POD1), and on postoperative day 7 (POD7) as well as the patient-reported time to recovery in days (days to return to daily activities). For each outcome, mean differences (MD) were estimated along with 95% confidence intervals (CIs) to compare the intervention and control groups. For the ecchymosis scores, standardized mean differences (SMD) were used due to variability in scoring scales across the studies. A *p*-value less than 0.05 was considered a statistically significant result.

A fixed-effect model was used in the absence of heterogeneity, while a random-effect model was applied in the presence of heterogeneity in the reported outcomes. The *I*^2^ test was used to assess heterogeneity. It was classified as low if less than 25%, moderate if between 25% and 50%, and high if over 50%. In case of high heterogeneity, a leave-one out sensitivity analysis was performed to identify and exclude the source of heterogeneity [28].

### Certainty of Evidence (GRADE)

The certainty of the generated evidence was evaluated according to the Grading of Recommendations, Assessment, Development and Evaluation (GRADE) criteria [29, 30]. The GRADE tool rates evidence into four levels of certainty: very low, low, moderate, and high. This evaluation considers several domains: risk of bias, inconsistency, indirectness, imprecision, publication bias, and other factors such as potential confounding.

## Results

### Literature Search

**Fig. 1:**
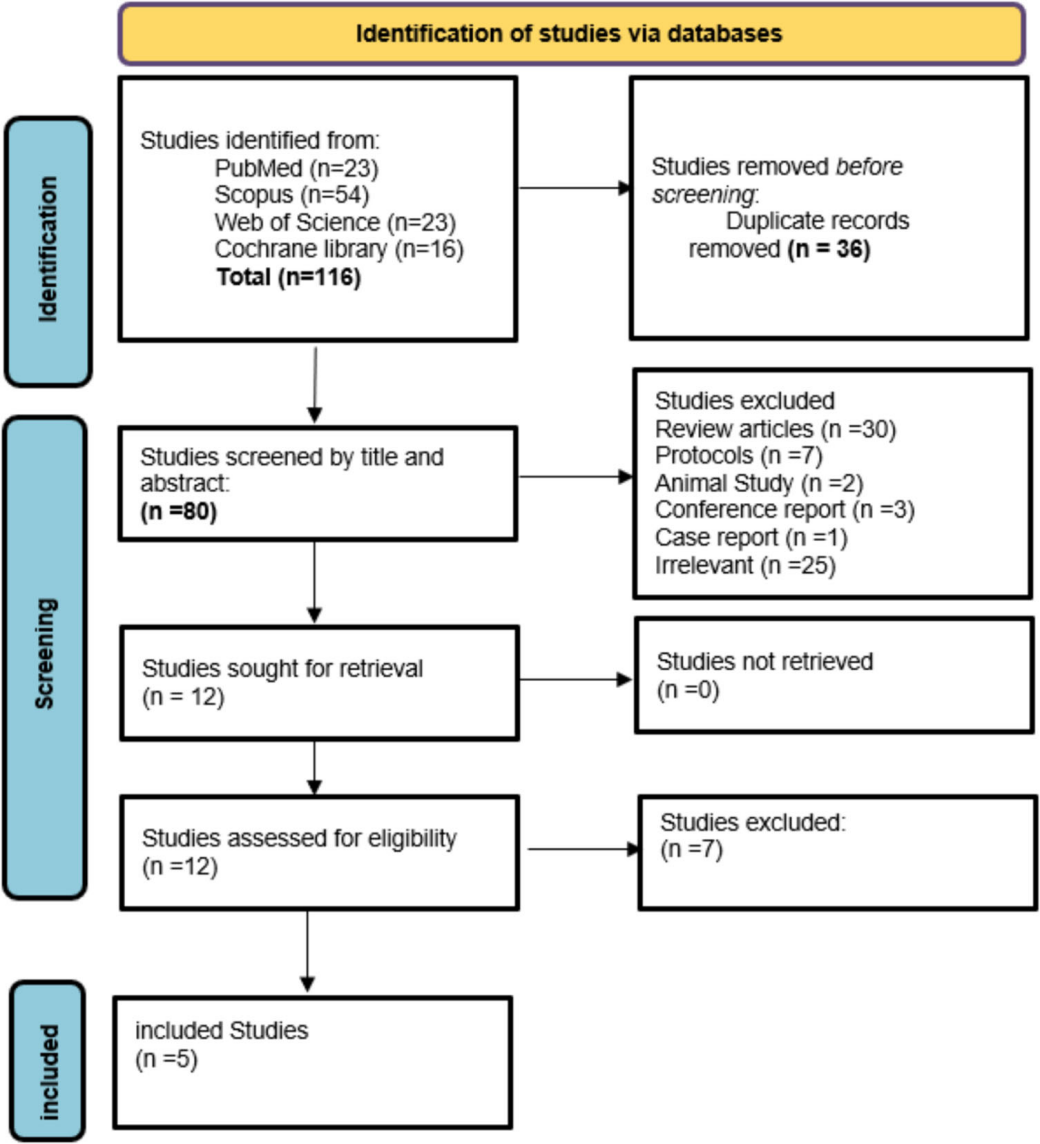
PRISMA flowchart

Through the systematic search, 116 articles were identified from several databases including PubMed (*n* = 23), Web of Science (*n* = 23), Cochrane Library (*n* = 16), and Scopus (*n* = 54) then the duplicates were identified and removed (*n* = 36). As a result, 80 articles were screened for their titles and abstracts, and 68 studies were found irrelevant and were excluded. Twelve articles were assessed for their full texts, as a result, seven studies were excluded. Finally, five studies were included in the analysis: Chaichumporn 2024 [31], Marous 2024 [4], Paramo 2024 [32], Sagiv 2018 [33], and Vogt 2024 [34]. The screening process is illustrated in the PRISMA flowchart. (Fig. 1).

### Characteristics of Included Studies

All five studies [4, 31–34] included in our analysis were randomized controlled trials, comprising a combined total of 594 participants (453 females, 76.3%) with a total of 1154 eyes. Marous 2024 [4], Paramo 2024 [32], and Vogt 2024 [34] were conducted in the USA while Chaichumporn 2024 [31] was conducted in Thailand, and Sagiv 2018 [33] was conducted in Israel. All studies used SC 50 mg/ml TXA except Vogt 2024 [33] used IV 1 g TXA and Marous 2024 [4] which used IV and SC TXA in separate groups. The SC TXA was injected in combination with 2% lidocaine and epinephrine (1:100,000) in all studies. While in Paramo [32] and Vogt [34] 0.5% bupivacaine was added.

Twenty-two patients were on aspirin, three were on Plavix (clopidogrel) and 19 were on anticoagulants. The time between TXA injection and the first incision ranged between 2 mins in Marous 2024 [4], ten minutes in both Chaichumporn 2024 [31] and Vogt 2024 [34] and at least 15 mins in Sagiv 2018 [33], while in Paramo 2024 [32] the first incision was made immediately after TXA injection.

The baseline characteristics of the included studies and participants are shown in Tables 1 and 2, respectively.

**Table 1:** Summary of the included studies

Study ID	Study design	Study arms		Other drugs taken during surgery	Distinct procedure	Time between injection and the first incision	Surgical technique	Country	Number of centers	Total Participants	Total eyes	Conclusion
		Intervention	Control									
Chaichumporn 2024	Double blinded, prospective randomized control trial	Subcutaneous 50 mg/ml tranexamic acid	Subcutaneous normal saline	2% lidocaine with epinephrine (1:100,000)	Upper eyelid blepharoplasty	10 minutes	NA	Thailand	one	15	30	TXA in lidocaine with epinephrine was found to increase intraoperative bleeding compared to lidocaine with epinephrine alone, but there was no difference in postoperative swelling or ecchymosis. TXA combined with lidocaine and epinephrine injected subcutaneously should be avoided until additional relevant data are obtained.
Marous 2024	Prospective, randomized, double-blinded, placebo controlled, comparative study	(Group 1) 1 g intravenous TXA OR (Group 2) 50 mg/ml subcutaneous TXA	(Group 3) No TXA	(Group 2 and 3) 2% lidocaine with epinephrine 1:100,000 mixed with normal saline 0.9% sodium chloride in a 1:1 ratio. (Group 3) 2% lidocaine with epinephrine 1:100,000 and TXA 100 mg/ml in a 1:1 ratio yielding and effective dose of 50 mg/ ml TXA bilaterally	Upper eyelid blepharoplasty	2 minutes	All skin incisions were made using a 15-blade, and excess skin and orbicularis were removed using Westcott scissors	USA	one	90	180	Preoperative TXA administered either IV or subcutaneously safely reduced postoperative ecchymosis and edema in patients undergoing upper eyelid blepharoplasty
Paramo 2024	Prospective, randomized, double-masked, controlled trial	Subcutaneous 50 mg/ml tranexamic acid	No TXA	(Group 1) 2.25 ml of 2% lidocaine with epinephrine, 2.25 ml of 0.5% bupivacaine with epinephrine, and 0.5 ml of TXA (Group 2) 2.5 ml of 2% lidocaine with epinephrine and 2.5 ml of 0.5% bupivacaine with epinephrine.	Upper and/or lower lid blepharoplasty or ptosis repair.	No time	NA	USA	two	130	260	Without a postinjection waiting period, SC TXA for eyelid surgery significantly decreased postoperative ecchymosis on postoperative day 0 and POW 1 but did not affect intraoperative bleeding.
Sagiv 2018	Prospective, randomized, double-blind, controlled trial	Subcutaneous 50 mg/ml tranexamic acid	Subcutaneous normal saline	A mixture of 2% lidocaine with either TXA 100 mg/mL or normal saline 0.9% sodium chloride (in a 1:1) mixture. Resulting in an effective 1% lidocaine as an anaesthetic agent in both groups and a TXA concentration of 50 mg/mL in the TXA group.	Upper eyelid blepharoplasty	15 minutes at minimum	Skin incisions were made using a no. 15 blade, excess skin was excised using a monopolar cautery	Israel	one	34	34	Subcutaneous TXA was associated with similar intra- and postoperative hemorrhage in upper eyelid blepharoplasty compared with placebo.
Vogt 2024	Randomized, double-blind, controlled trial	1 g intravenous TXA	No TXA	3 mL of local anesthetic mixture consisting of a 1:1 mixture of lidocaine with 1:100,000 epinephrine and 0.5% bupivacaine in each upper lid.	Upper eyelid blepharoplasty	10 minutes	Skin-only resection flap with scalpel and medial fat pad debulking	USA	two	325	650	Systemic tranexamic acid may reduce postoperative ecchymoses after upper blepharoplasty surgery, reaching significance at the eighth post-operative day, which may lead to improved patient satisfaction and decreased occupational downtime.

**Table 2:** Baseline characteristics of enrolled patients in the included studies

Study ID	Study arms	Age (years)	Sex (female), *N* (%)	Active smoking, *N* (%)	Using aspirin, *N* (%)	Using plavix, *N* (%)	Using anticoagulation, *N* (%)
Chaichumporn 2024		(30–45 years)—1 patient (46–60 years) —8 patients (>60 years)—6 patients	14 (93.3%)	NA	NA	NA	1 (6.7%)
Marous 2024	IV TXA	64.3 (7.25)	28 (93%)	2 (7%)	7 (23%)	0	8 (27%)
	SC TXA	66.6 (10)	29 (96%)	1 (4%)	4 (13%)	0	5 (17%)
	Non TXA	67.7 (7.5)	20 (67%)	3 (10%)	3 (10%)	2 (7%)	5 (17%)
Paramo 2024		67.9 (12.7)	107 (82.3%)	NA	NA	NA	NA
				NA	NA	NA	NA
Sagiv 2018	SC TXA	65 (8.5)	12 (71%)	3 (17.6%)	4 (23.5%)	1 (5.9%)	NA
	Saline	66 (6.25)	13 (76.5%)	3 (17.6%)	4 (23.5%)	0	NA
Vogt 2024	IV TXA	62	101 (73.1%)	NA	NA	NA	NA
	no TXA	64	129 (70%)	NA	NA	NA	NA

### Quality Assessment

The risk of bias assessment revealed that the included studies were all at low risk of bias. A summary of the risk of bias assessment domains is shown in Supplementary Figs. 1 and 2.

## Efficacy Outcomes

### Intraoperative Outcomes

#### Intraoperative Pain Score (VAS)

**Fig. 2:**
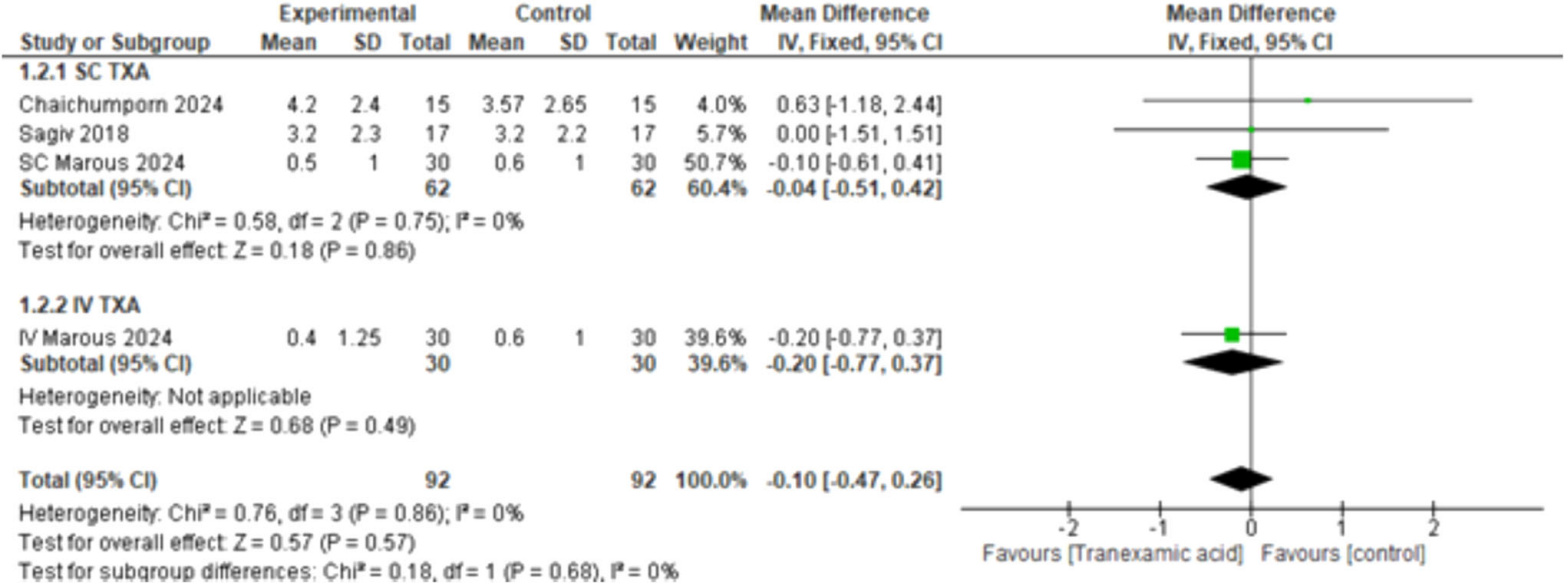
Intraoperative pain score (VAS)

The overall effect estimates showed no statistically significant difference between both groups (MD = – 0.10, 95% CI: [– 0.47 to 0.26], *p* < 0.57) and the combined results showed homogeneity (*p* < 0.86, *I*^2^ = 0%). The subgroups analysis showed that neither the SC nor IV routes of TXA showed significant results: SC TXA (MD = – 0.04, 95% CI: [– 0.51 to 0.42], *p* < 0.86), IV TXA (MD = – 0.20, 95% CI: [– 0.77 to 0.37], *p* < 0.49), with homogeneity detected among the subgroups (*p* = 0.68, *I*^2^ = 0%) (Fig. 2).

#### Operative Time in Minutes

**Fig. 3:**
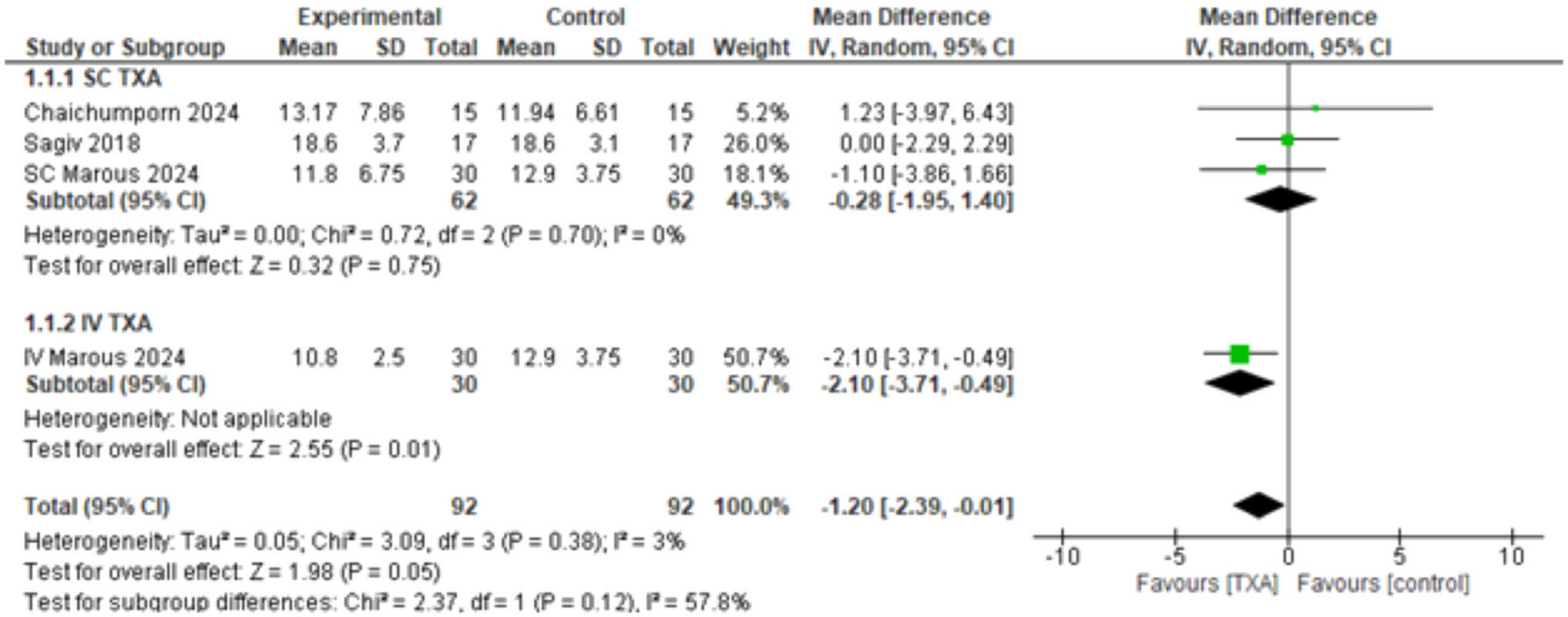
Operative time in minutes

The overall mean difference was statistically significant favoring TXA (MD = – 1.20, 95% CI: [– 2.39 to – 0.01], *p* < 0.05) and the combined results showed mild heterogeneity (*p* < 0.38, *I*^2^ = 3%). The subgroups analysis showed that the SC TXA did not have statistically significant different results (MD = – 0.28, 95% CI: [– 1.95 to 1.40], *p* < 0.75); however, IV TXA showed a statistically significant difference in favor of TXA (MD = – 2.10, 95% CI: [– 3.71 to – 0.49], *p* < 0.01), with high heterogeneity detected among the subgroups (*p* = 0.12, *I*^2^ = 57.8%) (Fig. 3). In order to solve the heterogeneity, a leave-one out sensitivity analysis was conducted, and it was best resolved by excluding Marous 2024 [4], as it is the only study in this analysis that used IV and not SC TXA. The overall effect estimates showed no statistically significant difference (MD = – 0.28, 95% CI: [– 1.95 to 1.40], *p* < 0.75) and the combined results showed homogeneity (*p* < 0.70, *I*^2^ = 0%). The subgroup analysis showed that SC TXA does not have statistically significant results (MD = – 0.28, 95% CI: [– 1.95 to 1.40], *p* < 0.75), while the IV TXA was not estimated (Supplementary Fig. 3).

#### Surgeon’s Assessment of Hemostasis (1–4 scale)

**Fig. 4:**
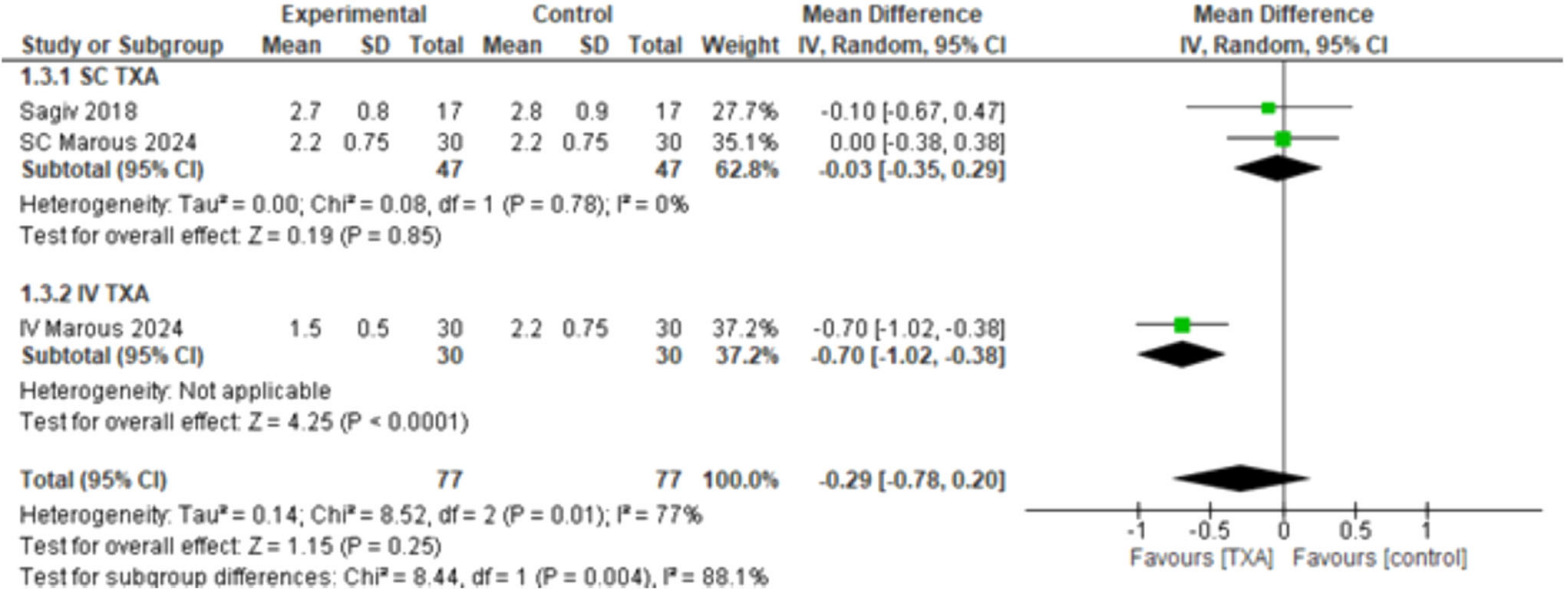
Surgeon’s assessment of hemostasis (1–4 scale)

The overall effect estimates showed no statistically significant difference (MD = – 0.29, 95% CI: [– 0.78 to 0.20], *p* < 0.25), and the combined results showed high heterogeneity (*p* < 0.01, *I*^2^ = 77%). The subgroup analysis showed that SC TXA does not have significant results (MD = – 0.03, 95% CI: [– 0.35 to 0.29], *p* < 0.85). On the other hand, IV TXA has a statistically significant difference in favor of the TXA (MD = – 0.70, 95% CI: [– 1.02 to – 0.38], *p* < 0.0001), with high heterogeneity detected among the subgroups (*p* =0.004, *I*^2^ = 88.1%) (Fig. 4). We performed a leave-one out sensitivity analysis to solve the heterogeneity, and it was best resolved by excluding Marous 2024 [4], as it is the only study in this analysis that used IV and not SC TXA. The overall effect estimates showed no statistically significant difference (MD = – 0.03, 95% CI: [– 0.35 to 0.29], *p* < 0.85). The subgroup analysis showed that SC TXA does not have significant results (MD = – 0.03, 95% CI: [– 0.35 to 0.29], *p* < 0.85), while the IV TXA was not estimated (Supplementary Fig. 4).

### Postoperative Outcomes

#### Ecchymosis Score According to the Time of Administration

**Fig. 5:**
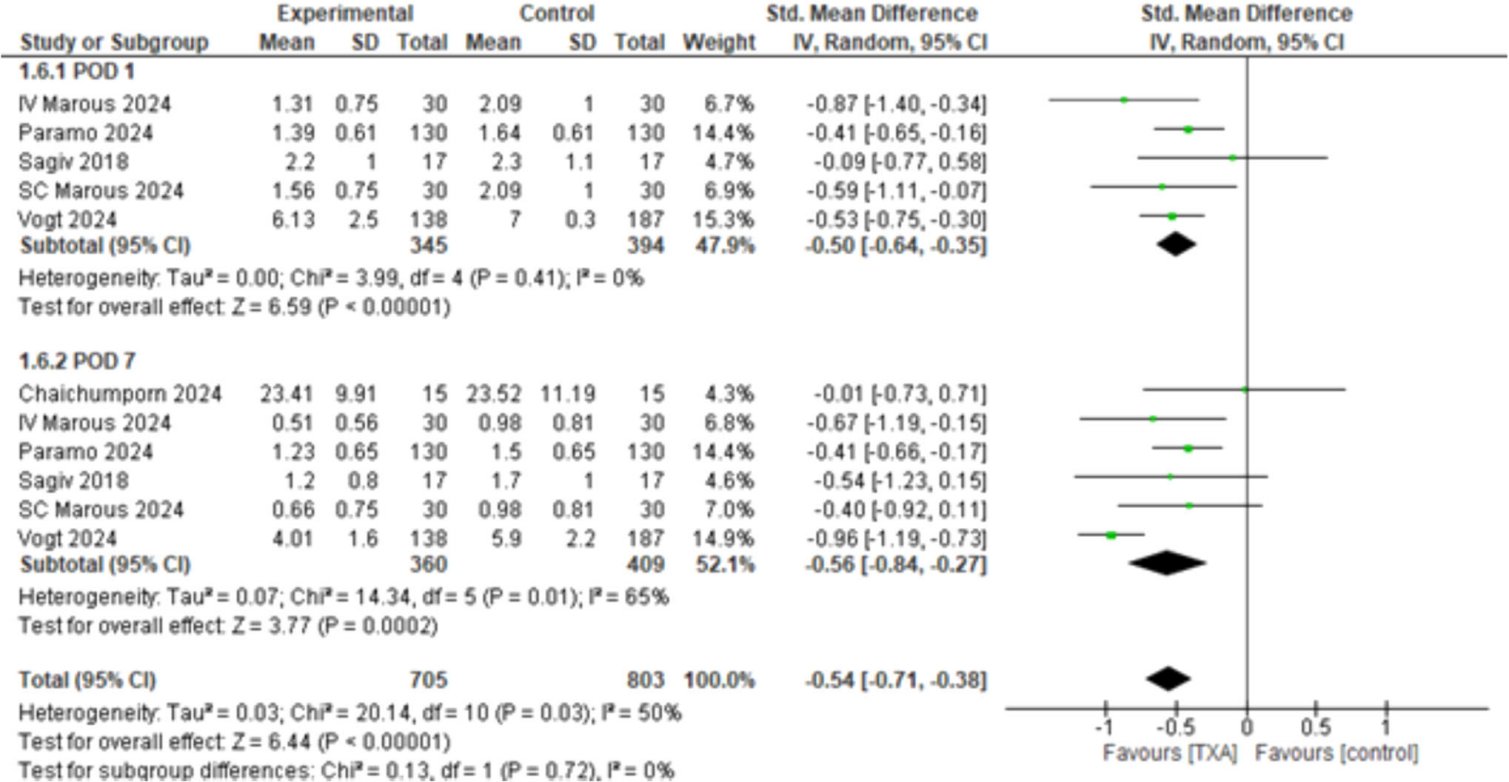
Ecchymosis score

The overall effect estimates showed a statistically significant difference favoring TXA (SMD = – 0.54, 95% CI: [– 0.71 to – 0.38], *p* < 0.00001) and the combined results showed mild heterogeneity (*p* < 0.03, *I*^2^ = 50%). The subgroups analyzing ecchymosis at POD1 and POD7 showed statistically significant results in both subgroups (SMD = – 0.50, 95% CI: [– 0.64 to – 0.35], *p* < 0.00001) and (SMD = – 0.56, 95% CI: [– 0.84 to – 0.27], *p* < 0.0002), respectively (Fig. 5). After applying the leave-one out sensitivity analysis, the heterogeneity was best resolved by removing Vogt 2024 [34]. The overall effect estimates showed statistically significant difference (SMD = – 0.47, 95% CI: [– 0.59 to – 0.35], *p* < 0.00001), and the combined results showed homogeneity (*p* < 0.69, *I*^2^ = 0%). The subgroup analysis showed that ecchymosis at POD1 and POD7 showed significantly better results favoring TXA when compared to the control (SMD = – 0.50, 95% CI: [– 0.64 to – 0.35], *p* < 0.00001), and (SMD = – 0.43, 95% CI: [– 0.62 to – 0.24], *p* < 0.00001), respectively. No heterogeneity was detected among the subgroups (*p* = 0.58, *I*^2^ = 0) (Supplementary Fig. 5). Furthermore, to make sure that the mixed-surgery population (Upper/Lower eyelid blepharoplasty and ptosis surgery) did not affect the robustness of our findings, we conducted a leave-one out sensitivity analysis to include only studies that performed upper eyelid blepharoplasty by excluding Paramo 2024 [32] as it is the only study the included patients performing upper or/and lower blepharoplasty or ptosis surgery. The overall effect estimates showed a statistically significant difference (SMD = – 0.59, 95% CI: [– 0.80 to – 0.39], *p* < 0.00001), and the combined results showed moderate heterogeneity of (*p* = 0.05, *I*^2^ = 48%). The subgroup analysis showed that ecchymosis at POD1 and POD7 still showed significantly better results favoring TXA when compared to the control (SMD = – 0.55, 95% CI: [– 0.75 to – 0.34], *p* < 0.00001), and (SMD = – 0.60, 95% CI: [– 0.93 to – 0.26], *p* = 0.0006) respectively, with no heterogeneity detected among the subgroups (*p* = 0.81, *I*^2^ = 0) (Supplementary Fig. 6). This concludes that the observed benefit of TXA in reducing ecchymosis is robust even when limited only to upper eyelid blepharoplasty.

#### Ecchymosis Score According to the Route of Administration

##### Ecchymosis Score at Postoperative Day One (POD1)

**Fig. 6:**
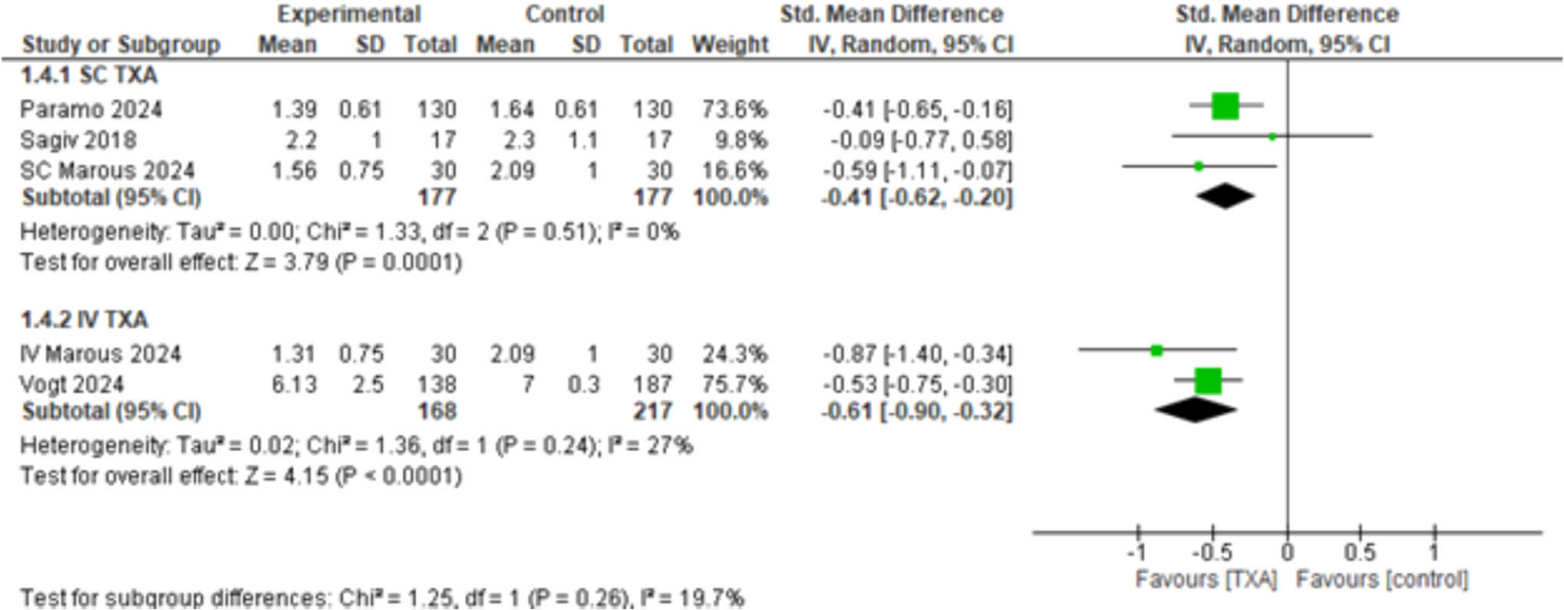
Ecchymosis score at postoperative day One (POD1)

Both SC and IV TXA showed significantly better results when compared to the control, (SMD = – 0.41, 95% CI: [– 0.62 to – 0.20], *p* < 0.0001), and (SMD = – 0.61, 95% CI: [– 0.90 to – 0.32], *p* < 0.0001) respectively, with mild heterogeneity detected among the subgroups (*p* = 0.26, *I*^2^ = 19.7%) (Fig. 6). After applying the leave-one-out sensitivity analysis excluding Paramo 2024 [32]. SC TXA became statistically non-significant (SMD = – 0.39, 95% CI: [– 0.87 to 0.09], *p* = 0.11). However, IV TXA still showed significantly better results when compared to placebo (SMD = – 0.61, 95% CI: [– 0.90 to – 0.32], *p* < 0.0001), with no heterogeneity among the subgroups (*p* = 0.44, *I*^2^ = 0%) (Supplementary Fig. 7).

##### Ecchymosis Score at Postoperative Day Seven (POD7)

**Fig. 7:**
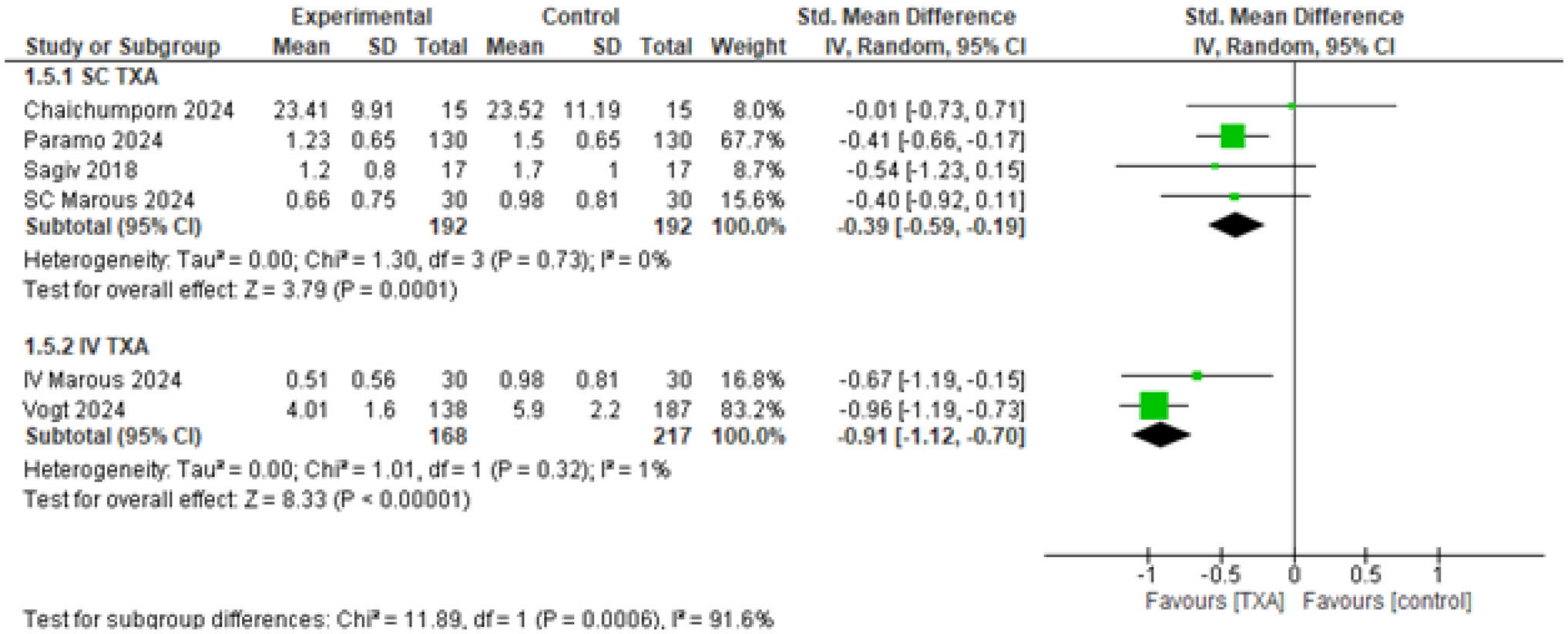
Ecchymosis score at postoperative day seven (POD7)

Both SC and IV TXA showed significantly better results when compared to the control, (SMD = – 0.39, 95% CI: [– 0.59 to – 0.19], *p* < 0.0001), and (SMD = – 0.91, 95% CI: [– 1.12 to – 0.70], *p* < 0.00001), with high heterogeneity detected among the subgroups (*p* = 0.0006, *I*^2^ = 91.6%) (Fig. 7). After applying the leave-one-out sensitivity analysis, the heterogeneity was best resolved by removing Vogt 2024 [34]. Both SC and IV TXA showed significantly better results when compared to the control (SMD = – 0.39, 95% CI: [– 0.59 to – 0.19], *p* < 0.0001), and (SMD = – 0.67, 95% CI: [– 1.19 to – 0.15], *p* < 0.01), respectively, with no heterogeneity among the subgroups (*p* = 0.33, *I*^2^ = 0%) (Supplementary Fig. 8). After applying the leave-one-out sensitivity analysis excluding Paramo 2024 [32]. SC TXA became statistically non-significant (SMD = – 0.34, 95% CI: [– 0.70 to 0.01], *p* = 0.06). However, IV TXA still showed significantly better results when compared to placebo (SMD = – 0.91, 95% CI: [– 1.12 to – 0.70], *p* < 0.00001), with substantial heterogeneity among the subgroups (*p* = 0.008, *I*^2^ = 86%) (Supplementary Fig. 9).

#### Patient’s Reported Time to Recovery in Days

**Fig. 8:**
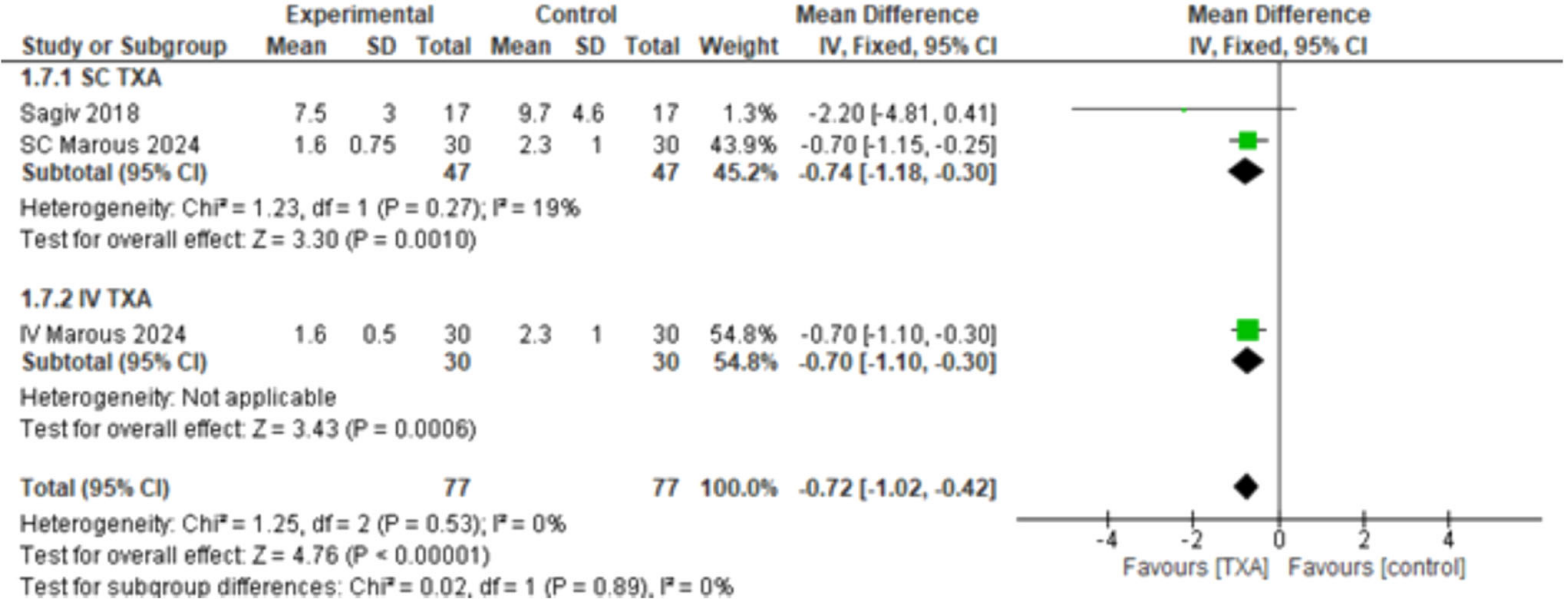
Patient’s reported time for recovery in days

The overall effect estimates showed a statistically significant difference in favor of TXA (MD = – 0.72, 95% CI: [– 1.02 to – 0.42], *p* < 0.00001) and the combined results showed homogeneity (*p* < 0.53, *I*^2^ = 0%). The subgroups analyzing the SC and IV routes of TXA favored both, SC TXA (MD = – 0.74, 95% CI: [– 1.18 to – 0.30], *p* < 0.0010), IV TXA (MD = – 0.70, 95% CI: [– 1.10 to – 0.30], *p* < 0.0006), with homogeneity detected among the subgroups (*p* = 0.89, *I*^2^ = 0%) (Fig. 8).

### GRADE Evidence

The quality of evidence regarding TXA efficacy in the most important outcomes versus control was assessed using GRADE. Intraoperative pain score, operative time, ecchymosis score, ecchymosis score POD1 and POD7 and patient’s reported time to recovery were not downgraded at any level and yielded high-quality evidence. The surgeon’s assessment of hemostasis was downgraded in the inconsistency domain. A summary of the findings and a GRADE evaluation of the outcomes are represented in Table 3.

**Table 3:** Summary of findings and quality of evidence

Outcome	Category	Studies (*N*)/eyes (*N*)	Estimate [95% CI]	Study limitations	Inconsistency	Indirectness	Imprecision	Publication bias	Certainty of evidence
Intraoperative pain score									
		3/244	MD = − 0.10 [− 0.47 to 0.26]	Fair quality	NS	Not serious^b^	Not serious	NA	⊕⊕⊕⊕ High
Operative time									
		3/244	MD = − 1.20 [− 2.39 to − 0.01]	Fair quality	NS	Not serious^b^	Not serious	NA	⊕⊕⊕⊕ High
Surgeon’s assessment of hemostasis									
		2/214	MD = − 0.29 [− 0.78 to 0.20]	Fair quality	Serious^a^	Not serious^b^	Not serious	NA	⊕⊕⊕◯ Moderate
Ecchymosis score									
		5/1154	MD = − 0.54 [− 0.71, − 0.38]	Fair quality	NA	Not serious^b^	Not serious	NA	⊕⊕⊕⊕ High
Ecchymosis score POD1									
		4/1124	MD = − 0.61 [− 0.90 to − 0.32]	Fair quality	NS	Not serious^b^	Not serious	NA	⊕⊕⊕⊕ High
Ecchymosis score POD7									
		5/1154	MD = − 0.91 [− 1.12 to − 0.70]	Fair quality	NS	Not serious^b^	Not serious	NA	⊕⊕⊕⊕ High
Patient’s reported time to recovery									
		2/214	MD = − 0.72 [− 1.02 to − 0.42]	Fair quality	NS	Not serious^b^	Not serious	NA	⊕⊕⊕⊕ High

*MD* Mean difference, *CI* Confidence interval, *NS* Not serious, *NA* Not applicable, *POD* Postoperative day

^a^*I*^2^ Greater than 50%, ^b^Drug and placebo interventions differed between RCTs

## Discussion

The current study is the first meta-analysis to assess the efficacy of TXA in blepharoplasty. Our findings suggest that TXA administration effectively reduces operative time and improves patient outcomes, as demonstrated by lower ecchymosis scores and a faster return to daily activities. Subgroup analysis indicated better outcomes with IV administration of TXA than SC, as reflected by lower ecchymosis scores and earlier recovery.

Laikhter et al. meta-analysis investigated the use of TXA in reducing hematoma formation in aesthetic plastic surgery and found that it led to a trend toward decreased hematoma incidence in such procedures [35]. This effect can be attributed to TXA's potent inhibition of fibrinolysis, which effectively stabilizes clot formation and decreases surgical trauma sequelae [36].

One of the major concerns regarding the use of TXA is the development of thromboembolic events as reported by Knowlton et al which found TXA to be associated with increased risks of embolism and mortality among trauma patients; however, TXA was not clearly established as an independent risk factor for thromboembolism [22]. Furthermore, a recent meta-analysis conducted by Taeuber et al found that the use of IV TXA was not associated with increased risks of thromboembolism regardless of the dose [37].

Chaichumporn et al. added lidocaine (2%) and epinephrine (0.001%) to both groups. In the TXA group, intraoperative bleeding was higher compared to lidocaine and epinephrine alone [31]. This discrepancy might be attributed to the time of the incision as the medication should ideally be given at least five to ten minutes before surgery. In addition, the acidity of TXA may reduce epinephrine's effectiveness by binding to either epinephrine molecules or vascular adrenergic receptors [38]. In contrast, Marous et al. and Paramo et al. also used lidocaine (2%) and epinephrine (0.001%) and showed that the TXA group exhibited significantly less intraoperative bleeding compared to the control [4, 32]. Although TXA can lead to thromboembolic events and seizures, all of the included studies in this meta-analysis reported no complications and no thromboembolic events throughout the follow-up period [4, 31–34, 39]. However, this risk profile underscores the need for shared decision-making between the surgeon and the patient. Marous et al. and Sagiv et al. included patients taking aspirin or anticoagulants in their studies and monitored them postoperatively with no complications reported in these cases [4, 33]. Furthermore, a recent meta-analysis recommended TXA use across a range of aesthetic plastic surgeries; however, it included only one study focused on blepharoplasty [33, 35]. It is also important to note that a study conducted by Sudhof et al [40] estimated that the cost of TXA is 37.80$ per 1 g in the USA and the cost of 100 mg/dl of IV TXA is around 48$.

The choice of surgical technique plays a role in intraoperative bleeding. Electrocautery allows for simultaneous dissection and hemostasis, potentially reducing bleeding compared to sharp scissors dissection, which may cause more bleeding but preserves tissue integrity. Similarly, skin-only excision typically results in less bleeding than combined skin-muscle excision, which involves deeper, more vascular tissue layers. Therefore, technique selection should be tailored to the clinical scenario, balancing hemostasis with surgical objectives.

This is the first systematic review and meta-analysis to comprehensively evaluate the effectiveness of TXA in blepharoplasty, offering a targeted assessment of its potential benefits.

While systematic reviews and meta-analyses of RCTs are considered the highest level of evidence, our study has certain limitations as significant heterogeneity was observed in certain outcomes which would compromise the reliability of the results. This heterogeneity could be explained by the relatively limited number of included studies in our analysis, which raises concerns about its comprehensiveness and introduces the risk of publication bias. In addition, the long-term effects of TXA were not assessed, which could provide valuable insights into both its sustained effectiveness and potential complications beyond the acute postoperative period. Furthermore, the interval between TXA administration and the first incision varies across the included studies, with one study reporting no time gap between these two events. Also, the heterogeneity could be attributed to the variations in the routes of administration (either IV or SC) and the concentration of the administrated TXA. Moreover, Paramo 2024 could have contributed to the heterogeneity as it is the only study that included upper/lower blepharoplasty and ptosis surgeries. Even though the remaining four studies were limited to upper blepharoplasty only, the exact approach was not specified (transcutaneous or transconjunctival) which would raise some concerns causing heterogeneity. Additionally, the safety outcomes of TXA were not comprehensively assessed, highlighting the need for further studies to explore this area. Collectively, these limitations highlight the necessity for studies that encompass a broader demographic and conduct detailed evaluations of long-term outcomes and safety profiles with possible adverse effects of both SC and IV TXA to better understand the true value of its use in blepharoplasty surgeries. Additionally, future studies should control the different surgical approaches of blepharoplasty to eliminate any confounding variables that would compromise the validity and generalizability of the findings.

A key limitation of this review is the uncertain clinical significance of the reported differences in operative and recovery times, as minimum important difference (MID) thresholds for these outcomes have not been established in the literature. Consequently, while statistical differences were identified, their practical relevance to patient care need further assessment. Such research is crucial for deepening our understanding of the role of TXA in blepharoplasty and its implications for patient care. Future research should aim to optimize the application of TXA in diverse patient populations and surgical settings, assess its long-term efficacy and safety, and evaluate its performance relative to other hemostatic approaches in blepharoplasty.

## Conclusion

TXA, particularly via IV administration, significantly improves intraoperative and postoperative outcomes in blepharoplasty by reducing ecchymosis, expediting recovery, and enhancing operative time and hemostasis. These findings support TXA’s use as an effective adjunct to improve blepharoplasty recovery, although further studies are warranted to optimize dosing and minimize thromboembolic risks.

## Supplementary Information

Below is the link to the electronic supplementary material.

Supplementary file1 (DOCX 291 kb)

## Data Availability

All data generated or analyzed during this study are included in this published article/as supplementary information files.
