# Supplementary material for: Evaluating Tranexamic Acid's Role in Upper Eyelid Blepharoplasty: A Systematic Review and Meta-Analysis
Source: Aesthetic Plast Surg. 2025 Oct 28;49(23):6416–30. doi: 10.1007/s00266-025-05294-w (PMC12738644; doi:10.1007/s00266-025-05294-w)
Supplement: Supplementary file 1 — Supplementary file1 (DOCX 291 kb) [file 266_2025_5294_MOESM1_ESM.docx]

**Evaluating Tranexamic Acid's Role in upper eyelid Blepharoplasty: A Systematic Review and Meta-Analysis**

Mohamed Abo Zeid^1*^, Kareem Khalefa^1^, Mohamed Al Diab Al Azzawi^2^, Amr Elrosasy^3^, Amr M. Abou Elezz^1^, Mohamed S. I. Mohamed^4^, Richard C. Allen^5^, Hashem Abu Serhan^6^

1. Faculty of Medicine, Tanta University, Tanta, Egypt; [Mohamadaboelyazeed0@gmail.com](mailto:Mohamadaboelyazeed0@gmail.com), [kareemkhalefa188@gmail.com](mailto:kareemkhalefa188@gmail.com), [Amr_31060929@med.tanta.edu.eg](mailto:Amr_31060929@med.tanta.edu.eg)
2. Faculty of Medicine, National Ribat University, Khartoum, Sudan; [moh.fares69@gmail.com](mailto:moh.fares69@gmail.com)
3. Faculty of Medicine, Cairo University, Cairo, Egypt; [10912022103193@stud.cu.edu.eg](mailto:10912022103193@stud.cu.edu.eg)
4. College of Medicine, QU Health, Qatar University, Doha, Qatar; [mm1907261@qu.edu.qa](mailto:mm1907261@qu.edu.qa)
5. Department of Ophthalmology, Dell Medical School, University of Texas, Austin, TX United States; richardcutlerallen@gmail.com
6. Department of Ophthalmology, Hamad Medical Corporation, Doha, Qatar; [Habuserhan@hamad.qa](mailto:Habuserhan@hamad.qa)

****Corresponding author:***

**Name: Mohamed Abo Zeid
 E-mail:** [**Mohamed_31059599@med.tanta.edu.eg**](mailto:Mohamed_31059599@med.tanta.edu.eg) **Phone: +02 01125313213**

**ORCID:** [**https://orcid.org/0009-0007-8165-9470**](https://orcid.org/0009-0007-8165-9470)


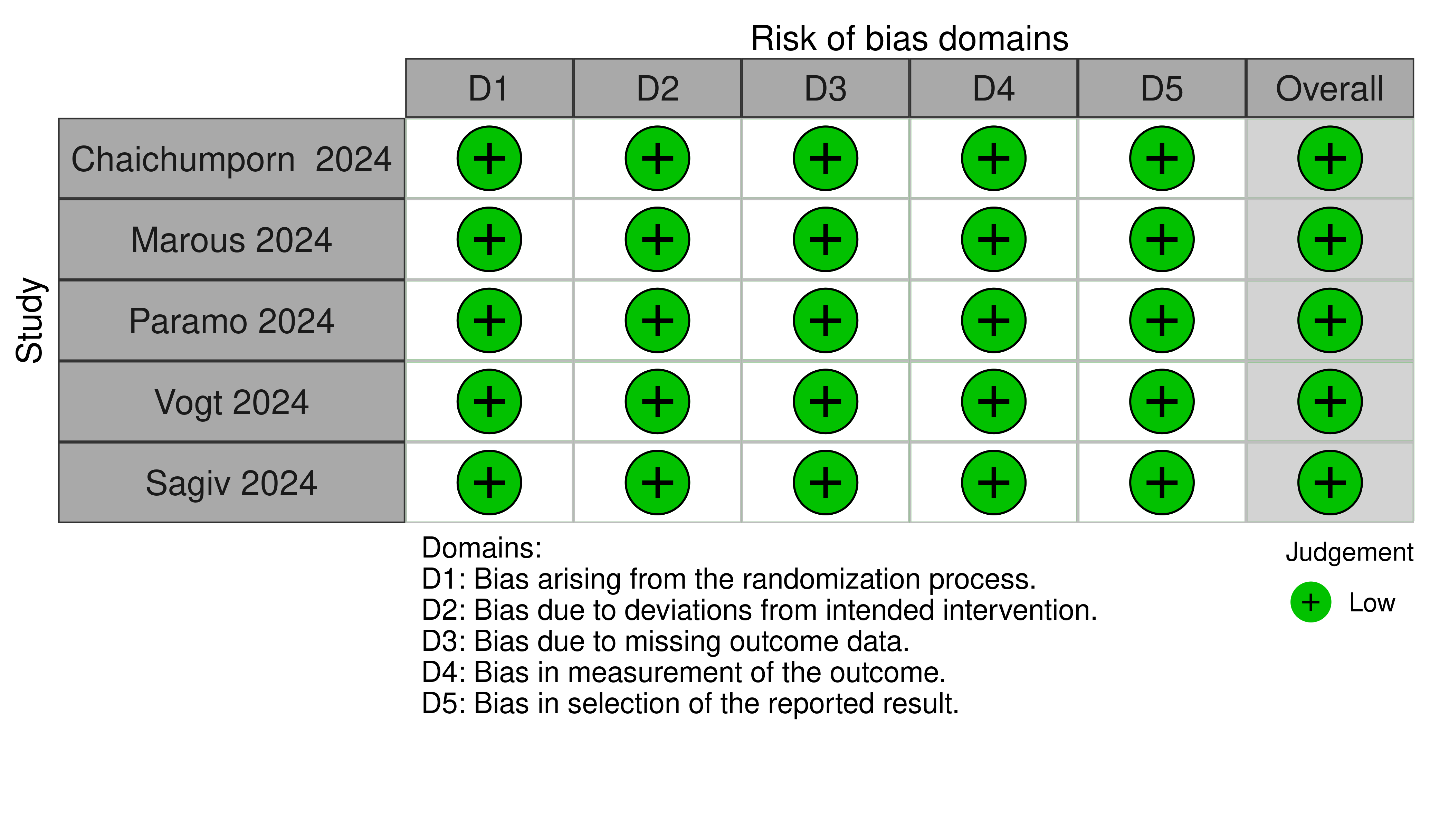
**(Supplementary Figure 1).** The bias-risk assessment diagram of the included articles**.**
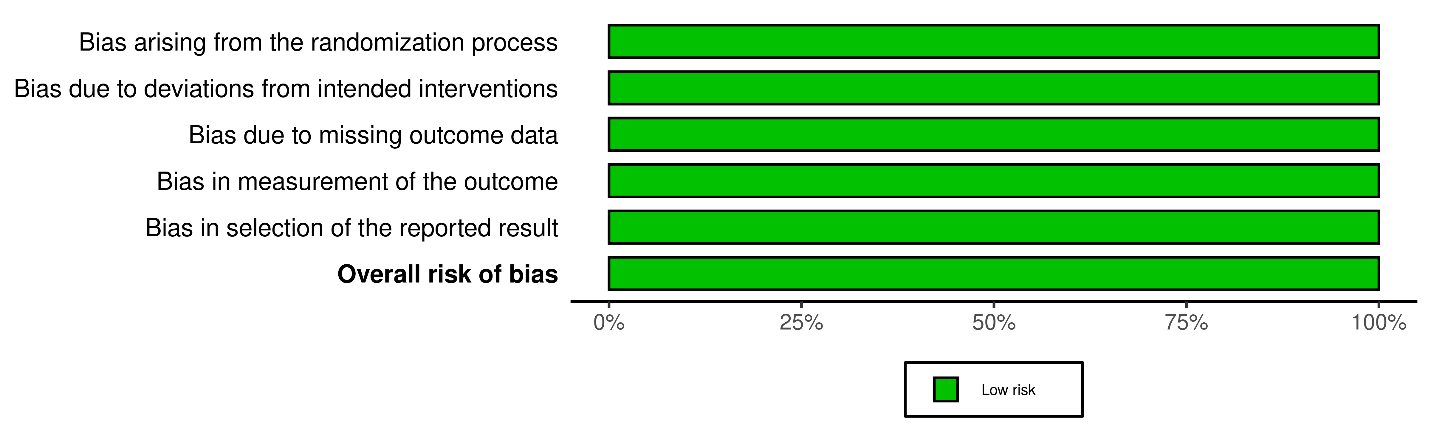
 **(Supplementary Figure 2).** The bias evaluation bar graph of the included articles.**
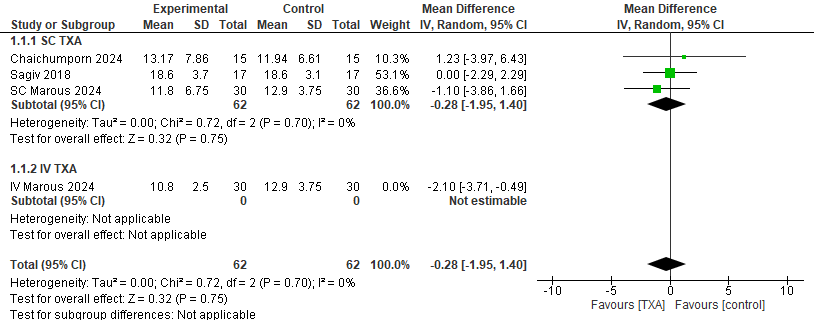
 (Supplementary Figure 3).** Operative time in minutes with leave-one out sensitivity analysis (After removing Marous 2024)

**
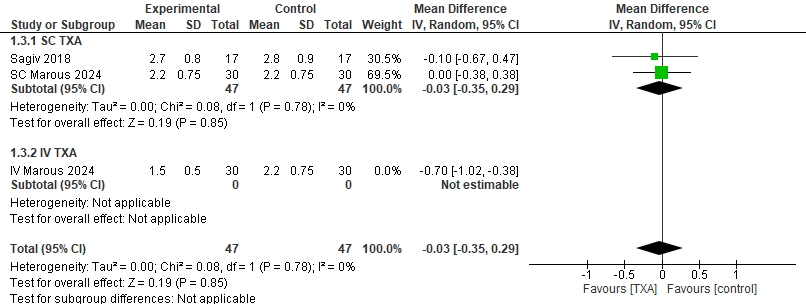
 (Supplementary Figure 4).** Surgeon’s assessment of hemostasis (1-4 scale) with leave-one out sensitivity analysis (After removing Marous 2024)

**
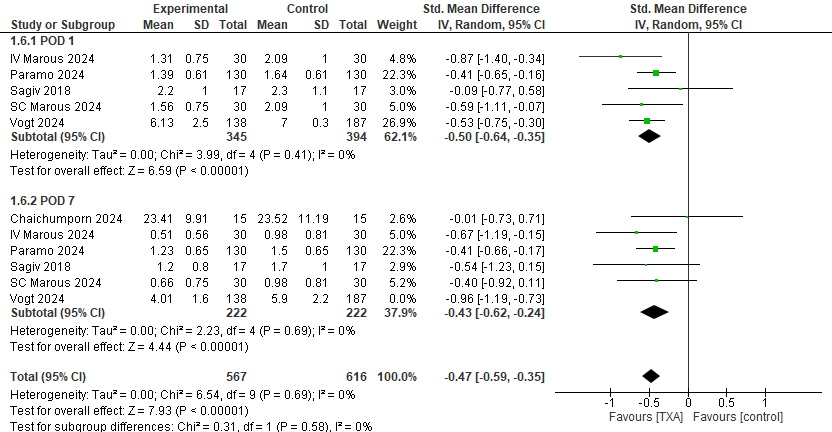
 (Supplementary Figure 5).** Ecchymosis score with leave-one out sensitivity analysis (After removing Vogt 2024)


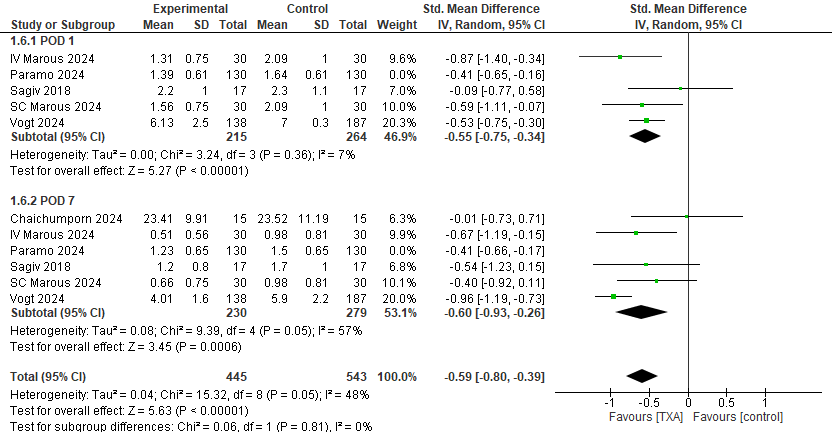


**(Supplementary Figure 6).** Ecchymosis score with leave-one out sensitivity analysis (After removing Paramo 2024)

**
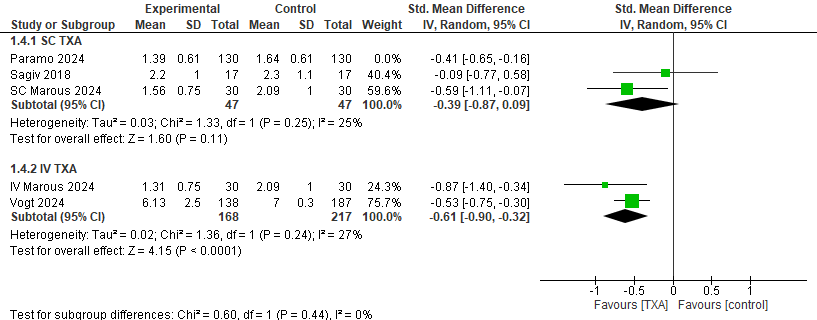
 (Supplementary Figure 7).** Ecchymosis score POD1 with leave-one out sensitivity analysis (After removing Paramo 2024)

**
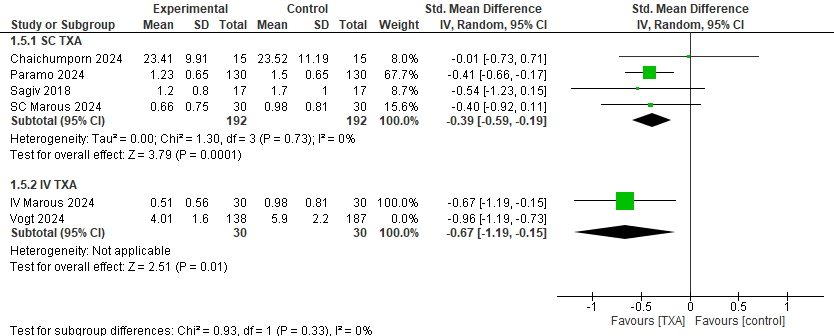
 (Supplementary Figure 8).** Ecchymosis score POD7 with leave-one out sensitivity analysis (After removing Vogt 2024)

**
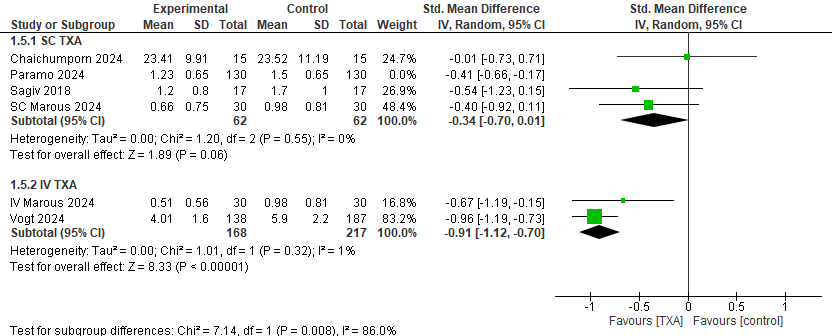
 (Supplementary Figure 9).** Ecchymosis score POD7 with leave-one out sensitivity analysis (After removing Paramo 2024)
